# Effect of MgO Additive on Properties of Corundum–Mullite Duplex Ceramic Synthesized from High-Alumina Fly Ash

**DOI:** 10.3390/ma18204805

**Published:** 2025-10-21

**Authors:** Suwei Dai, Xiaowen Wu, Bingcheng Luo

**Affiliations:** 1 College of Science, China Agricultural University, Beijing 100083, China; 2511110618@stu.pku.edu.cn; 2 School of Materials Science and Technology, China University of Geosciences Beijing, Beijing 100083, China; xwwu@cugb.edu.cn

**Keywords:** fly ash, ceramics, mechanical properties, microstructure, mullite

## Abstract

Corundum–mullite duplex ceramics were fabricated via a solid-state reaction technique using high-alumina fly ash and α-alumina powders. The effects of magnesium oxide on bulk density, apparent porosity, compressive strength, and microstructure of the duplex ceramics were investigated by scanning electronic microscopy, mechanical testing, and X-ray diffraction, respectively. Results showed that the prepared ceramics were mainly dominated by mullite and corundum phases, and the mullite was in the form of columns and crosses to form a net-like structure. The bulk density and the compressive strength increased with the increase in MgO addition, while the porosity decreased contrariwise. Optimal performance among tested compositions was observed at 12 wt% MgO addition, yielding a bulk density of 3.012 g·cm^−3^, a porosity of 8.12%, and a compressive strength of 263 MPa, demonstrating the potential of this composite for high-performance ceramic applications.

## 1. Introduction

As environmental concerns escalate, the focus on repurposing industrial waste materials has intensified. Fly ash, a byproduct of raw coal combustion in power plants and industrial facilities, poses significant storage and environmental challenges in many countries. However, it is also recognized as one of the world’s most abundant raw material resources [1,2]. With its high content of silicon dioxide and alumina, fly ash exhibits excellent strength and refractoriness after high-temperature sintering. Moreover, the new phase (e.g., mullite.) will be synthesized while sintered at the proper temperature due to the chemical reaction reported in the previous literature [3]. Consequently, converting fly ash into a high-value charged material presents a promising strategy for its utilization [4].

Researchers have explored various applications of fly ash as a raw material, including the preparation of mullite, zeolite, sialon, and cement [5,6]. Amrhein et al. [4] developed a method to fabricate the high-charge zeolitic material from fly ash by heating the fly ash in strong base (>0.5 M) for a few days. Park et al. [7] fabricated mullite whiskers through the recycling of coal fly ash. Ojha et al. [8] synthesized X-type zeolite from coal fly ash using an alkali fusion and hydrothermal treatment process, achieving a maximum surface area of 383 m^2^·g for the product, with the crystallinity of the zeolite varying with fusion temperature and peaking at 823 K. Kudyba-Jansen et al. [9] produced Ca-α/β-sialon ceramics via carbothermal reduction and nitridation, utilizing fly ashes as sources, and observed enhanced density (91 to 99%) and mechanical properties (strength increased from 340 to 550 MPa) with the addition of sintering additives like CaO. Guerrero et al. [10] reported the synthesis of belite cement clinker from coal fly ash of high Ca content via the hydrothermal-calcination route of the fly ash without the need of extra additives.

Fly ash is increasingly recognized as a cost-effective resource for the ceramics, environmental protection, and construction industries, offering a solution to environmental challenges while also generating high-value materials. Furthermore, there has been significant interest in the development of composite materials using fly ash as a filler [11,12,13,14,15]. Moutsatsou et al. [13] fabricated Al/fly ash metal matrix composites using the powder metallurgy technique and showed that the incorporation of high-Ca fly ash into the metal matrix increased the amount of Ca–Si phases produced, leading the harder surface of the composites. Raghavendra et al. [11] synthesized the polyaniline/fly ash conducting composites with fly ash as filler by using an in situ polymerization method. Daoud et al. [12] developed 4032–10 vol.% fly ash particle composite foams with two types of fly ash particles, observing that the modulus of elasticity of the 4032–fly ash precipitator composite exceeded that of the unreinforced foam by more than eightfold. Gu et al. [14] created epoxy composites filled with varying volumes of fly ash, noting that the tangent delta (tan δ) values peaked at the glass transition temperatures for composites with 30–50 vol% fly ash, with a gradual decrease as frequency increased. Woszuk et al. [15] utilized fly ash as a cost-effective filler in asphalt mixtures, which improved the water and frost resistance of the asphalt.

Mullite is distinguished by its remarkable properties, including a high melting point, excellent creep resistance, superior high-temperature strength, and outstanding chemical stability under harsh conditions [16]. The 3:2 mullite structure is highly stable and capable of accommodating a significant number of transition metal ions as solid solutions [17]. Recently, multiphase ceramics, including corundum–mullite ceramics, have garnered considerable attention. This duplex ceramic combines the strengths of corundum and mullite, offering superior mechanical strength, good wear resistance, low thermal conductivity, and excellent high-temperature resistance [18]. However, the preparation of corundum–mullite ceramic requires high temperature, and the resulting mechanical properties may not always meet engineering standards. Extensive efforts have been directed towards incorporating additives such as Y_2_O_3_, V_2_O_5_, CeO_2_, TiO_2_, ZrO_2_, Ho_2_O_3_, and MgO into ceramics to significantly reduce sintering temperatures and enhance product performance [19,20,21,22]. Li et al. [19] investigated the impact of V_2_O_5_ on the mechanical properties of mullite ceramics synthesized from fly ash and bauxite, revealing a flexural strength of 108 MPa with 10% V_2_O_5_ at 1500 °C. Li et al. [23] found that V_2_O_5_ promotes the in situ growth of spearhead columnar mullite due to the increased liquid phase during sintering, achieving compressive strengths of 16.8 MPa and thermal conductivities of 1.04 W m^−1^·K^−1^ in porous corundum–mullite ceramics. Kong et al. [24] introduced MnO_2_ as a sintering aid to corundum–mullite ceramics, with the formation of secondary phases and solid solution strengthening enhancing density and strength. Feng et al. [25] elucidated the strengthening mechanism of MnO_2_ by characterizing the ionic valence of elements, proposing that the formation of Mn^2+^ leads to a distorted corundum lattice, facilitating rod-like mullite formation. Xu et al. [26] added Sm_2_O_3_ to cordierite–mullite–corundum composite ceramics to bolster thermal shock resistance and decrease thermal conductivity. Prusty et al. [22] examined the influence of MgO on the structural, microstructural, and hardness properties of zirconia mullite, demonstrating that MgO not only stabilizes the cubic zirconia phase but also aids in the formation of cross-linked mullite grains. Dong et al. [16] fabricated mullite ceramics from recycled fly ash and bauxite with MgO addition, with thermal analysis, bulk density, and pore structure indicating that MgO promotes sintering and improves mechanical strength. Nevertheless, the effect of MgO on the performance of corundum–mullite duplex ceramics has not been extensively studied.

In this work, we report on the preparation, microstructure, and properties of corundum–mullite duplex ceramic. The duplex ceramic was fabricated by a solid-state method with high-alumina fly ash and α-Al_2_O_3_ powders (Al:Si = 3:2) as raw materials. MgO was employed to improve the performance of the product. The effects of MgO addition on the mechanical properties, phase composition, and microstructure of the duplex ceramic was investigated.

## 2. Materials and Methods

**Figure 1:**
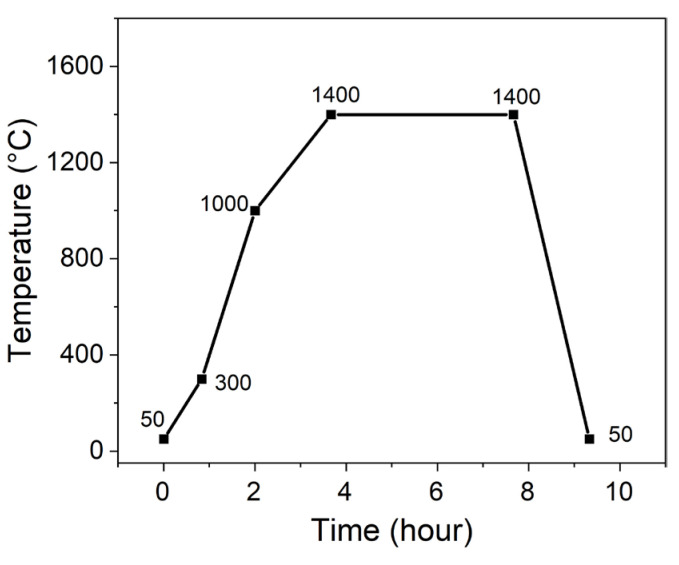
Sintering curves of fly ash-based corundum–mullite ceramics.

All samples were prepared by solid-state reaction method with fly ash and α-Al_2_O_3_ powders as raw material. The fly ash was provided by a power plant in the Ningxia Hui Autonomous Region, China, and the α-Al_2_O_3_ powders were purchased from Henan Jiyuan Brother Material Co., Ltd., Jiyuan, Henan, China. The polyvinyl alcohol and MgO were chosen as agglomerant and additive, respectively, which were purchased from Beijing Jingwen Chemical Reagents Company, Beijing, China. The chemical composition of fly ash is shown in Table 1. The mixture of fly ash and α-Al_2_O_3_ powders was based on the molar ratio 3:2 of Al to Si, to which 0, 3, 6, 9, and 12 wt% MgO were added with respect to the total amount of fly ash and α-Al_2_O_3_. Then, the whole mixture was dry mixed by drilling for 24 h using a planetary ball mill. Next, 3~5 wt% polyvinyl alcohol was added to pelletize, after which the samples were formed by dry extrusion molding using hydraulic machine at 8 MPa. Subsequently, the pellets were sintered in electric resistance furnace at 1400 °C for 4 h, and then cooled naturally within the furnace. The sintering temperature schedule is shown in Figure 1. In the end, the corundum–mullite ceramic composites were prepared.

The apparent porosity and density of the samples were measured by Archimedes drainage method according to the standard test method [27], which were calculated by the following formula:

*V* = π*bd*^2^/4 (1)

*ρ*_b_ = *m*_0_/*V* (2)

*ω* = (*m*_s_ − *m*_0_)/(*m*_s_ − *m*_f_) × 100% (3)

*ρ*_s_ = *ρ*_0_ × *m*_0_/(*m*_s_ − *m*_0_) (4)

where *b* and *d* are the thickness (mm) and diameter (mm) after sintered, respectively, *ω* is the apparent porosity, *V* is the volume (mm^3^) after sintered, *m*_0_, *m*_s_, and *m*_f_ are dry weight (g), wet weight (g), and float weight (g), respectively, *ρ*_b_, *ρ*_s_, and *ρ*_0_ are the sample bulk density (kg·m^−3^), sample true density (kg m^−3^), and water density (kg m^−3^), respectively. The compression tests were carried out at room temperature on a Reger Universal Testing Machine (Shenzhen Reger Instrument Co., Ltd., Shenzhen, China). The applied cross-head speed was 0.5 mm min^−1^. The stress and strain were calculated by

*σ* = *F*/*A* (5)

*ε* = Δ*l*/*l*_0_ (6)

where *σ* is the stress (MPa), *ε* is the strain, *F* is the load (N) at yield, *A* is the cross-section area (mm^2^), *l_0_* is the original length (mm) of specimen, Δ*l* is the sample displacement (mm). All the results were calculated based on the average of five tests.

The microstructure of all the samples was obtained using the Field Emission Scanning Electron Microscope FESEM/EDS (LEO-1530, Carl Zeiss SMT AG, Oberkochen, Germany). Since the ceramics are electrically non-conducting, they were coated with gold–palladium alloy of 15~20 nm thickness using a sputter coater prior to scanning electron microscopy (SEM) examination. The room temperature X-ray diffractograms of all the samples were recorded using X-ray diffractometer (Rigaku D/max2200, Tokyo, Japan) with Cu Kα radiation (λ = 0.15406 nm). The X-ray powder diffraction patterns were recorded in the angular range of 10–70° with a step size of 0.02° using monochromatic X-rays.

In the finite element analysis (FEA), a Voronoi tessellation structure (65 mm × 120 mm) was implemented through coding, with corresponding material properties assigned to the grain boundaries and grains, respectively. A high-precision mesh was generated for the model. The alumina–mullite ceramic grains were assigned a Young’s modulus of 80 GPa, a density of 1.935 g cm^−3^, and a Poisson’s ratio of 0.23. The sapphirine phase was assigned a Young’s modulus of 300 GPa, a Poisson’s ratio of 0.29, and a density of 3.45 g cm^−3^. The mechanical properties at the grain boundaries were set to be 10% lower than those of the grain interiors. Complete fracture of the ceramic was assumed to occur when the cumulative energy release rate, G, exceeded the critical energy release rate, Gc. The Gc value was set to 40 J m^−2^ for the alumina–mullite ceramic and 10 J m^−2^ for the sapphirine. A localized pressure was applied to the top surface of the ceramic model to simulate fracture. The corresponding load-displacement curve was obtained through post-processing of the simulation data.

## 3. Results and Discussion

### 3.1. Phase Analysis of the Ceramic Composites

**Figure 2:**
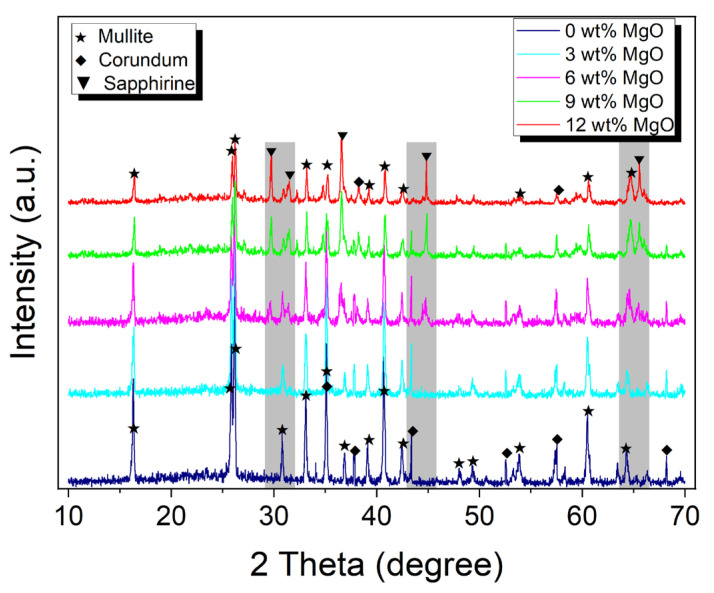
XRD patterns of fly ash-based corundum–mullite ceramics with MgO contents (0, 3, 6, 9, and 12 wt%). The shadows indicate the range sapphirine appearing.

Figure 2 shows the X-ray diffraction patterns of corundum–mullite ceramic samples synthesized from high-alumina fly ash with MgO addition of 0, 3, 6, 9, and 12 wt%. As shown in powder XRD patterns, all the reflection peaks for total samples with 0–12 wt% MgO addition corresponding to mullite and corundum are clearly seen, in excellent agreement with the Powder Diffraction File (PDF) # 83-1881 and PDF # 74-1081 from the Joint Committee on Powder Diffraction Standards (JCPDS), respectively. This reveals that corundum–mullite diphase ceramic materials are successfully prepared from high-alumina fly ash.

The observations indicate a marked difference in intensity between the mullite and corundum phases, with mullite exhibiting a considerably stronger presence. This suggests that mullite is the predominant crystalline phase, while corundum assumes a secondary role. In samples containing 0–3 wt% MgO, the crystalline phases are exclusively composed of corundum and mullite. At a 6 wt% MgO content, the sapphirine phase begins to manifest subtly, as indicated in the shadow in Figure 2. This phase corresponds to a rare light blue or green aluminum–magnesium silicate mineral, occurring in the proximity of the corundum and mullite phases. As the MgO content is increased to the range of 9–12 wt%, the sapphirine phase becomes more distinct, aligning with the description provided in PDF # 19-0750. Figure 2 also reveals a trend where the peak intensities of both mullite and corundum diminish with the progressive addition of MgO, while the intensity of the sapphirine phase incrementally rises. This shift could be attributed to the formation of (Mg,Al)_8_(Al,Si)_6_O_20_ sapphirine crystals, a result of devitrification of the MgO-containing aluminum silicate liquid by a solution-reprecipitation process during cooling [28]. Table 2 shows the calculated lattice parameters of corundum–mullite ceramic samples with varying MgO content. The data illustrate that the lattice constant b_0_ for the mullite phase increases, in contrast to the decrease observed in both a_0_ and c_0_. Concurrently, the lattice constant a_0_ for the corundum phase exhibits a gradual decline with the incremental addition of MgO. These findings underscore the substantial impact of MgO content on the unit cell dimensions of the ceramic phases.

### 3.2. Microstructure of the Corundum–Mullite Ceramics

**Figure 3:**
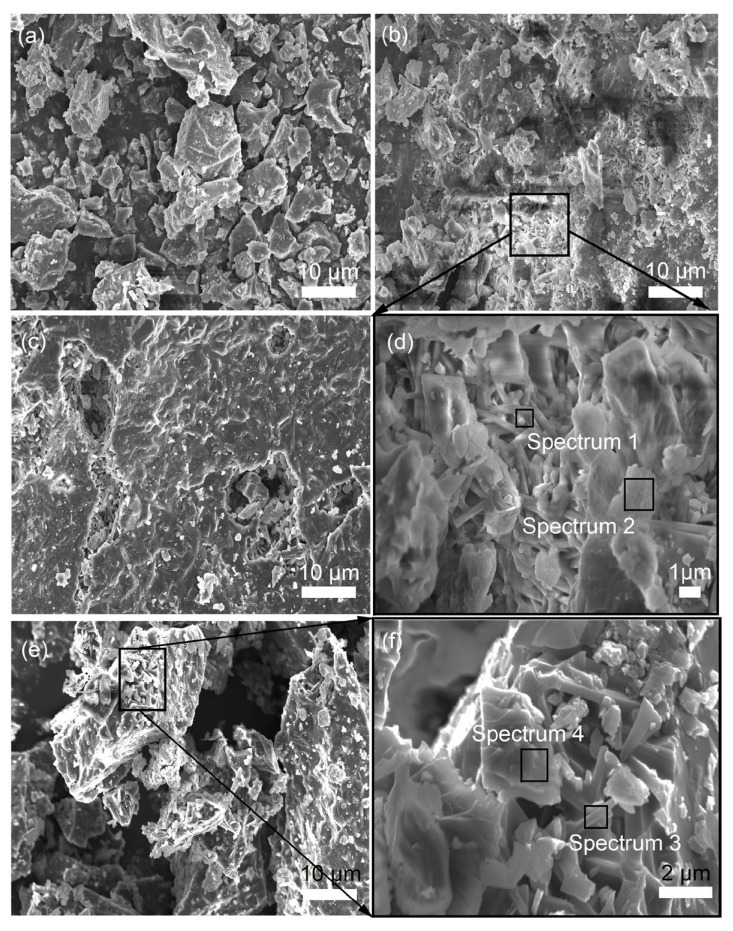
SEM images of fracture surfaces of samples sintered at 1400 °C with MgO of 0 wt% (**a**), 3 wt% (**b**,**d**), 6 wt% (**c**), and 9 wt% (**e**,**f**).

The SEM images in Figure 3 reveal the morphological characteristics of samples sintered at 1400 °C with varying MgO contents ranging from 0 to 9 wt%. As shown in Figure 3b,d and Table 3, the addition of 3 wt% MgO modifies the morphology of preexisting mullite and corundum phases. The mullite crystals adopt a columnar structure, measuring approximately 2 to 4 μm in length and 0.3 to 0.5 μm in width, yielding a length-to-diameter ratio of roughly 4 to 13. In contrast, the corundum phase presents itself in an irregular morphology. With an increase in MgO content to 6 wt%, as shown in Figure 3c, the quantity of columnar mullite crystals proliferates. This growth is attributed to the increased liquid phase resulting from the higher MgO content, which facilitates the expansion of short columnar mullite. This process leads to the cracking of cross-linked networks, enabling the absorption of Al_2_O_3_ and SiO_2_ from the surrounding high-temperature aluminosilicate liquid phase. Consequently, the mullite crystals further develop into refined columnar aggregates along the C-axis [29]. These aggregates then intertwine to form a networked structure, thereby enhancing the density and mechanical strength of the corundum–mullite ceramics. The role of MgO in this context is primarily as a sintering aid, facilitating the formation of interlocked mullite grains [22]. The calculated lattice parameters in Table 2 and SEM images co-confirm selective dissolution of smaller mullite grains. As illustrated in Figure 3e,f, when the MgO addition reaches 9 wt%, the abundance of the corundum phase further decreases, in alignment with the XRD analysis results presented in Figure 2. At this MgO concentration, the irregularly shaped corundum is observed to be closely associated with the columnar mullite, as depicted in Figure 3f. Meanwhile, the pore-filling effect is primarily driven by the liquid phase with the addition of MgO, and interlocking of large mullite columns reduces intergranular pores.

### 3.3. Mechanical Properties of the Corundum–Mullite Ceramics

**Figure 4:**
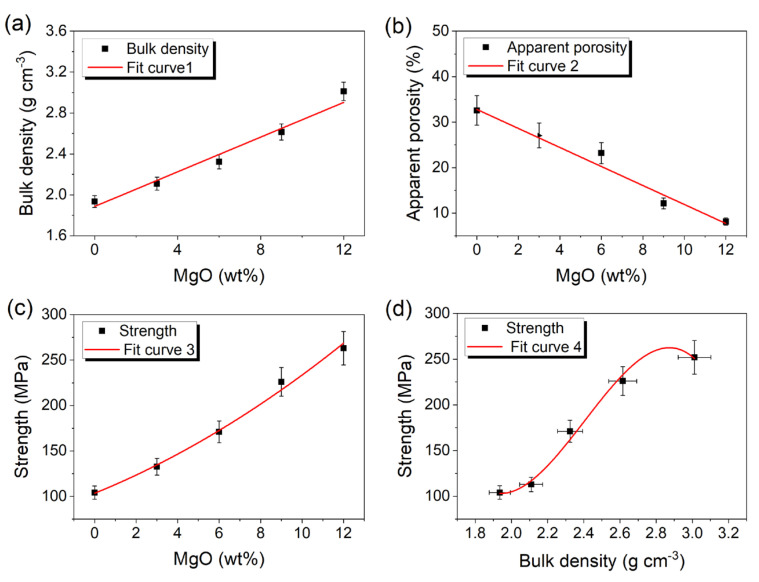
The variation in bulk density (**a**), apparent porosity (**b**), compressive strength (**c**) with MgO contents (0, 3, 6, 9, and 12 wt%) and strength versus bulk density (**d**) of fly ash−based corundum−mullite ceramics.

The physical attributes of fly ash-derived corundum–mullite ceramics, incorporating MgO contents of 0, 3, 6, 9, and 12 wt%, are depicted in Figure 4. This figure elucidates the trends in bulk density, apparent porosity, and compressive strength as a function of MgO content. As illustrated in Figure 4a–c, an increase in MgO content is correlated with a rise in bulk density and compressive strength, accompanied by a corresponding decrease in porosity. This pattern of bulk density variation with MgO content aligns with the findings reported by Dong et al. [16]. At a 12 wt% MgO addition, the ceramic sample achieves a bulk density of 3.012 g cm^−3^, a porosity of 8.12%, and a compressive strength of 263 MPa (Figure 4a–c). It is evident that the incorporation of MgO has a beneficial effect on the Al_2_O_3_-SiO_2_ binary system. As the MgO content increases, the sintering temperature is reduced, and a greater number of Al_2_O_3_-SiO_2_-MgO ternary liquid phases emerge. The rise in liquid phases facilitates grain diffusion and pore filling, as evidenced by the XRD analysis (Figure 2) and the ceramic morphology (Figure 3). The cubic polynomial trend (R^2^ = 0.96) highlights the direct relationship between densification and mechanical enhancement (Figure 4d). Consequently, the addition of MgO not only increases density and reduces porosity but also significantly enhances the compressive strength of the ceramics.

### 3.4. FEA of the Corundum–Mullite Ceramics

**Figure 5:**
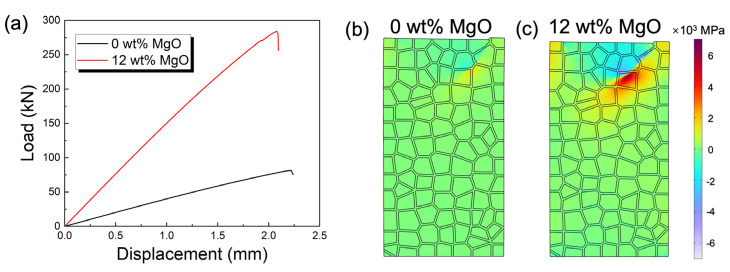
(**a**) Load−displacement curve during compressive measurement of corundum−mullite ceramics at room temperature. Finite element simulation of pressure distribution under maximum load in 0 wt% MgO addition (**b**) and 12 wt% MgO (**c**) addition in fly ash-based corundum−mullite ceramics.

To deepen the understanding of the compressive fracture mechanism of magnesia-doped corundum–mullite dual-phase ceramics, FEA was employed to simulate the load-displacement curve, as shown in Figure 5a. In Figure 5b, we first simulated the pressure distribution at the maximum compressive strength for pure corundum–mullite ceramics. Subsequently, a portion of the corundum–mullite grains was replaced with newly generated sapphirine to represent the corundum–mullite ceramics with 12 wt% MgO addition, as illustrated in Figure 5c. The finite element model of the ceramic grains was locally loaded until fracture occurred. In the case of 0 wt% MgO (Figure 5a), the Young’s modulus reached approximately 38 MPa. With the addition of 12 wt% MgO, the system’s Young’s modulus significantly increased to about 143 MPa. This enhancement in resistance to deformation originates from the superior mechanical properties of the sapphirine formed in the system with 12 wt% MgO addition. Along with the increased density and reduced porosity (Figure 4a,b), the system with 12 wt% MgO addition achieved an approximately 3.7-fold improvement under the maximum load. Although this is slightly higher than the experimental improvement of 2.5 times, it still reflects that the addition of MgO can lead to a substantial enhancement in the mechanical properties of the corundum–mullite system.

## 4. Conclusions

In this work, corundum–mullite duplex ceramic was prepared by solid-state reaction method from high-alumina fly ash and α-alumina powders. X-ray diffraction analysis confirmed the formation of the desired mullite and corundum phases. Subsequently, the addition of MgO as a sintering aid was aimed at enhancing the mechanical properties of the ceramics. The intensity of the mullite and corundum peaks decreased with increasing MgO content, while the sapphirine phase became more distinct. SEM imaging revealed that the mullite phase assumed a columnar structure, forming an interconnected network within the ceramic matrix. The physical properties of the ceramics were significantly influenced by the MgO additive, leading to an increase in bulk density and compressive strength, and a reduction in porosity. At a 12 wt% MgO addition, the corundum–mullite duplex ceramic achieved a bulk density of 3.012 g cm^−3^, an apparent porosity of 8.12%, and a remarkable compressive strength of 263 MPa. The finite element simulation results of the pressure distribution under the maximum load show good agreement with the experimental results. This work demonstrates that the incorporation of MgO as a sintering aid is an effective and practical approach to produce fly ash-based ceramics with superior mechanical attributes.

## Figures and Tables

**Table 1:** Chemical composition of fly ash.

Oxides	SiO_2_	TiO_2_	Al_2_O_3_	Fe_2_O_3_	FeO	MgO	CaO	MnO	K_2_O	P_2_O_5_
Ratio (wt%)	46.83	1.12	37.05	6.93	0.00	0.66	2.78	0.05	0.54	0.2

**Table 2:** Calculated lattice parameters of fly ash-based corundum–mullite ceramics with different MgO contents (0, 3, 6, 9, and 12 wt%).

Samples	MgO (wt%)	Mullite				Corundum	
		a_0_/Å	b_0_/Å	c_0_/Å	V/Å^3^	a_0_/Å	V/Å^3^
No. 1	0	7.574	7.715	2.898	169.3	5.137	85.100
No. 2	3	7.567	7.725	2.897	169.3	5.135	85.010
No. 3	6	7.556	7.729	2.892	168.9	5.135	85.080
No. 4	9	7.552	7.733	2.889	168.7	5.114	84.710
No. 5	12	7.504	7.766	2.888	168.3	—	—

**Table 3:** Average composition of the detected elements in the samples with 3 wt% and 9 wt% addition of MgO.

	Element (in wt%)				
	O	Mg	Al	Si	Others
With 3 wt% MgO					
Spectrum-1	42.06	1.26	21.59	17.72	17.37
Spectrum-2	46.75	3.55	26.09	12.87	10.74
With 9 wt% MgO					
Spectrum-3	43.19	1.62	18.34	22.43	14.42
Spectrum-4	47.55	4.23	25.40	18.07	4.75

## Data Availability

The original contributions presented in the study are included in the article, further inquiries can be directed to the corresponding author.
